# Lung impedance changes during awake prone positioning in COVID-19. A non-randomized cross-over study

**DOI:** 10.1371/journal.pone.0299199

**Published:** 2024-02-21

**Authors:** Jacob Rosén, Peter Frykholm, Malin Jonsson Fagerlund, Mariangela Pellegrini, Francesca Campoccia Jalde, Erik von Oelreich, Diddi Fors

**Affiliations:** 1 Department of Surgical Sciences, Anaesthesiology and Intensive Care Medicine, Uppsala University, Uppsala, Sweden; 2 Perioperative Medicine and Intensive Care, Karolinska University Hospital, Solna, Sweden; 3 Section of Anesthesiology and Intensive Care Medicine, Department of Physiology and Pharmacology, Karolinska Institutet, Solna, Sweden; 4 Section of Thoracic Anesthesiology and Intensive Care, Department of Molecular Medicine and Surgery, Karolinska Institutet, Solna, Sweden; CHU Nantes, FRANCE

## Abstract

**Background:**

The effects of awake prone positioning (APP) on respiratory mechanics in patients with COVID-19 are not well characterized. The aim of this study was to investigate changes of global and regional lung volumes during APP compared with the supine position using electrical lung impedance tomography (EIT) in patients with hypoxemic respiratory failure due to COVID-19.

**Materials and methods:**

This exploratory non-randomized cross-over study was conducted at two university hospitals in Sweden between January and May 2021. Patients admitted to the intensive care unit with confirmed COVID-19, an arterial cannula in place, a PaO_2_/FiO_2_ ratio <26.6 kPa (<200 mmHg) and high-flow nasal oxygen or non-invasive ventilation were eligible for inclusion. EIT-data were recorded at supine baseline, at 30 and 60 minutes after APP-initiation, and 30 minutes after supine repositioning. The primary outcomes were changes in global and regional tidal impedance variation (TIV), center of ventilation (CoV), global and regional delta end-expiratory lung-impedance (dEELI) and global inhomogeneity (GI) index at the end of APP compared with supine baseline. Data were reported as median (IQR).

**Results:**

All patients (n = 10) were male and age was 64 (47–73) years. There were no changes in global or regional TIV, CoV or GI-index during the intervention. dEELI increased from supine reference value 0 to 1.51 (0.32–3.62) 60 minutes after APP (median difference 1.51 (95% CI 0.19–5.16), p = 0.04) and returned to near baseline values after supine repositioning. Seven patients (70%) showed an increase >0.20 in dEELI during APP. The other EIT-variables did not change during APP compared with baseline.

**Conclusion:**

Awake prone positioning was associated with a transient lung recruiting effect without changes in ventilation distribution measured with EIT in patients with hypoxemic respiratory failure due to COVID-19.

## Introduction

Awake prone positioning (APP) of non-intubated, spontaneously breathing patients with hypoxemia was introduced early and adopted widely during the Corona virus disease 2019 (COVID-19) pandemic aiming to delay, or avoid intubation in a setting of resource shortage [1–4]. A large randomized controlled trial including 1126 patient reported that APP reduced a composite outcome of intubation rate and mortality in patients with hypoxemic respiratory failure due to COVID-19 [5]. Later, meta-analyses confirmed that APP reduces intubation rates but has little or no effect on mortality [6, 7]. Patients with oxygenation improvement during APP are often defined as responders by both bedside clinicians and in published reports [8]. However, this is not associated with improved long-term outcomes [9, 10] and warrants further investigation of novel tools for selection of patients who may benefit from APP.

Although the effects of prone positioning on lung physiology are well established among intubated patients with moderate to severe acute respiratory distress syndrome (ARDS) [11], they have not been thoroughly investigated during APP in patients with respiratory failure [12]. Specifically, few studies describe the effects of APP on global and regional ventilation in patients with hypoxemic respiratory failure due to COVID-19 [13–18].

Electrical impedance tomography (EIT) is a non-invasive bedside device for the continuous monitoring of lung volumes [19]. By electrodes equally spaced and circumferentially attached to the chest surface, EIT measures changes in thoracic electrical conductivity throughout the breathing cycle [20]. Variation of impedance closely correlates to changes in global and regional lung volume, which has been validated through comparisons with several other techniques [21–27].

In one case report of a COVID-19 patient, end-expiratory lung impedance (EELI) measured by EIT increased during APP [13]. As EELI correlates to end-expiratory lung volume [27] this was interpreted as alveolar recruitment and suggested to contribute to the observed coincident improvement in oxygenation. Recently a physiological study of fifteen patients with hypoxemic respiratory failure showed that EELI increased during APP although no sub-analysis was performed for the eight patients with COVID-19 in this cohort [17]. The aim of this study was to investigate lung volume changes during APP compared with the supine position using EIT in patients with hypoxemic respiratory failure due to COVID-19.

## Materials and methods

### Study design, setting and ethical statement

This was an exploratory interventional non-randomized unblinded cross-over study conducted between 4 January and 23 May 2021 in two intensive care units (ICU) at the Uppsala University Hospital and the Karolinska University Hospital in Sweden. The study was performed in compliance with the 1964 Helsinki declaration and Good Clinical Practice. The Transparent Reporting of Evaluations with Nonrandomized Designs (TREND) guideline was followed for reporting (S1 Checklist). The protocol was registered at the ISRCTN registry (ISRCTN54918435) 15 June 2020 (S1 File). The study was performed as a sub study of the previously published PROFLO trial [28]. However, after the inclusion of two patients for EIT-measurements, the main trial was terminated early due to futility in February 2021 and the remainder of the patients in the current investigation were included separately. Ethical approval was provided by the Swedish Ethical Review Authority 10 June 2020 (2020–02743) with amendments approved 29 December 2020 (2020–06425) and 16 April 2021 (2021–00856) for inclusion at both hospitals and separate from the main trial respectively. Written informed consent was obtained from all participants.

### Patients

Adult patients (≥18 years) admitted to the ICU with COVID-19 confirmed by positive SARS-CoV-2 reverse transcription polymerase chain reaction tests, an arterial cannula in place as part of their clinical management, a PaO_2_/FiO_2_ ratio <26.6 kPa (<200 mmHg) and high-flow nasal oxygen (HFNO) or non-invasive ventilation (NIV) for respiratory support were eligible for inclusion. Exclusion criteria were inability to assume prone position for at least one hour, contraindication to EIT (presence of implanted active devices such as pacemakers, or skin damage interfering with EIT electrode placement), hemodynamic or respiratory instability requiring emergent interventions, previous intubation for COVID-19 pneumonia, pregnancy, terminal illness with less than one year life expectancy, do-not-intubate decision, or inability to understand oral or written study information. The research team screened patients admitted to the ICU and approached eligible patients for informed consent.

### Study protocol

Before study protocol initiation, a mandatory supine positioning session of at least 60 minutes was conducted to attenuate the effects on lung volume distribution from previous body positions. An elastic EIT thoracic band of appropriate size containing equally spaced electrodes was subsequently placed around the thorax at the level of the 4^th^-6^th^ intercostal space and adequate signal quality was confirmed. The bed was adjusted to a 10 degree head up incline which was maintained throughout the protocol. Flow was set to at least 50 L min^-1^ and PEEP to at least 5 cm H_2_O for patients with HFNO and NIV respectively. For patients with NIV, inspiratory pressure support was set by the treating physician and was not changed. FiO_2_ was set to reach an SpO_2_ of 92–96%. After initial titration, all settings were fixed during the protocol unless desaturation <90% for more than 5 min, or <85% for more than 1 minute occurred, in which case the FiO_2_ was increased as necessary to reach the target SpO_2_. After baseline measurements in the supine position, patients were turned to the prone position for 60 minutes. Semi-prone positions were not allowed. After prone positioning, patients were positioned supine for 30 more minutes. All measurements were recorded at baseline in the supine position (T1), after 30 (T2) and 60 minutes (T3) in the prone position and 30 minutes after supine repositioning (T4).

### Data collection and measurements

Information regarding patient characteristics, comorbidities, laboratory results and medication was extracted from the electronic medical record at each study site.

EIT-measurements were obtained with a Pulmovista 500 (Dräger, Lübeck, Germany) at the Uppsala University Hospital and a Swisstom-Sentec (Swisstom AG-Sentec, Landquart, Switzerland) at the Karolinska University Hospital. EIT-data were recorded with a frame rate of 20Hz for at least five minutes at each pre-specified data collection time point. EIT-data were stored, and EIT-images reconstructed off-line. Twenty respiratory cycles were averaged per timepoint and analyzed using EITdiagn version 1.6 (Dräger, Lübeck, Germany) for data recorded with the Pulmovista 500 and ibeX version 1.5 (Swisstom AG-Sentex, Landquart, Switzerland) for data recorded with the Swisstom-Sentec. Frame rate resampling and low-pass filters were not used. EIT-images were analyzed and horizontally divided into four equally sized regions of interest (ROI) in the anteroposterior axis: ventral, mid-ventral, mid-dorsal and dorsal ROI. These definitions of lung-regions did not change during APP and consequently, ventral during APP was towards the bed. The following EIT-variables were analyzed:

1. Global and regional tidal impedance variation (TIV) which closely correlates with tidal volume [21, 29]. TIV data are presented relative to the reference measurement at baseline (T1) which was 100 for all patients.
2. Center of ventilation (CoV) expressed as percent of the chest diameter and describes the gravitation point of ventilation. CoV was derived analyzing the tidal impedance distribution in 20 small equally sized ROIs spanning the image in the anteroposterior direction [30, 31]. CoV >50% indicates that the gravitation point of ventilation is towards the ventral regions of the lung (towards the bed in APP).
3. Delta end-expiratory lung impedance (dEELI) reflecting the change in end-expiratory lung volume compared with T1 (baseline reference value at T1 for all patients: 0) [27].
4. Global inhomogeneity (GI) index which reflects the variation of ventilation distribution within the lung. It is calculated as the sum of the absolute difference between the median value and every pixel value in each EIT-image and normalized to the sum of the impedance values within the lung area [32, 33]. This may be expressed as a formula:
$$GI=\frac{\sum_{x,y\epsilon lung}\left\lceil {DI}_{xy}-Median({DI}_{lung})\right\rceil }{\sum_{x,y\epsilon lung}{DI}_{xy}}$$
  where DI is the value of the differential impedance in the tidal images, DI_*xy*_ is the pixel in the identified lung area and DI_*lung*_ is all the pixels representing the lung area (adapted from Zhao et al. [32]).

Arterial blood gas samples were analyzed with ABL 800 flex (Radiometer, Brønshøj, Denmark). Respiratory rate, heart rate and invasive arterial blood pressure were recorded at each predefined data collection timepoint.

### Outcomes

The primary outcome variables were global and regional TIV, CoV, global and regional dEELI and GI index at T3 compared with T1. Secondary outcomes were changes in respiratory rate, SpO_2_, PaO_2_/FiO_2_ ratio, PaCO_2_, heart rate, systolic, diastolic, and mean arterial blood pressure. We analyzed correlation between change in dEELI and PaO_2_/FiO_2_ ratio from T1 to T3.

### Sample size calculation

As no previous studies on EIT-variables during APP in patients with moderate to severe hypoxemic respiratory failure due to COVID 19 were available, we aimed to perform an exploratory study. Thus, no sample size calculation was performed. The ethical permission granted the inclusion of 60 patients. However, due to rapidly declining case numbers, the study ended after the inclusion of eleven patients in May 2021.

### Statistical methods

Statistical analyses were performed using Base SAS 9.4. Descriptive statistics were reported as median (interquartile range) or number (percentage) as appropriate. The Wilcoxon signed rank test was used to test non-normally distributed data for differences in measurements between timepoint T3 and T1. Because of the small sample size and the exploratory nature of this study, we did not perform multiple testing of all timepoints. Correlation was analyzed using the Spearman rank correlation coefficient. Effect sizes for non-normally distributed data were estimated with the distribution-free method using order statistics and reported as median differences with 95% confidence intervals (CI). Two-sided p-values of <0.05 were considered significant. No post-hoc correction for multiple testing was performed.

## Results

**Fig 1:**
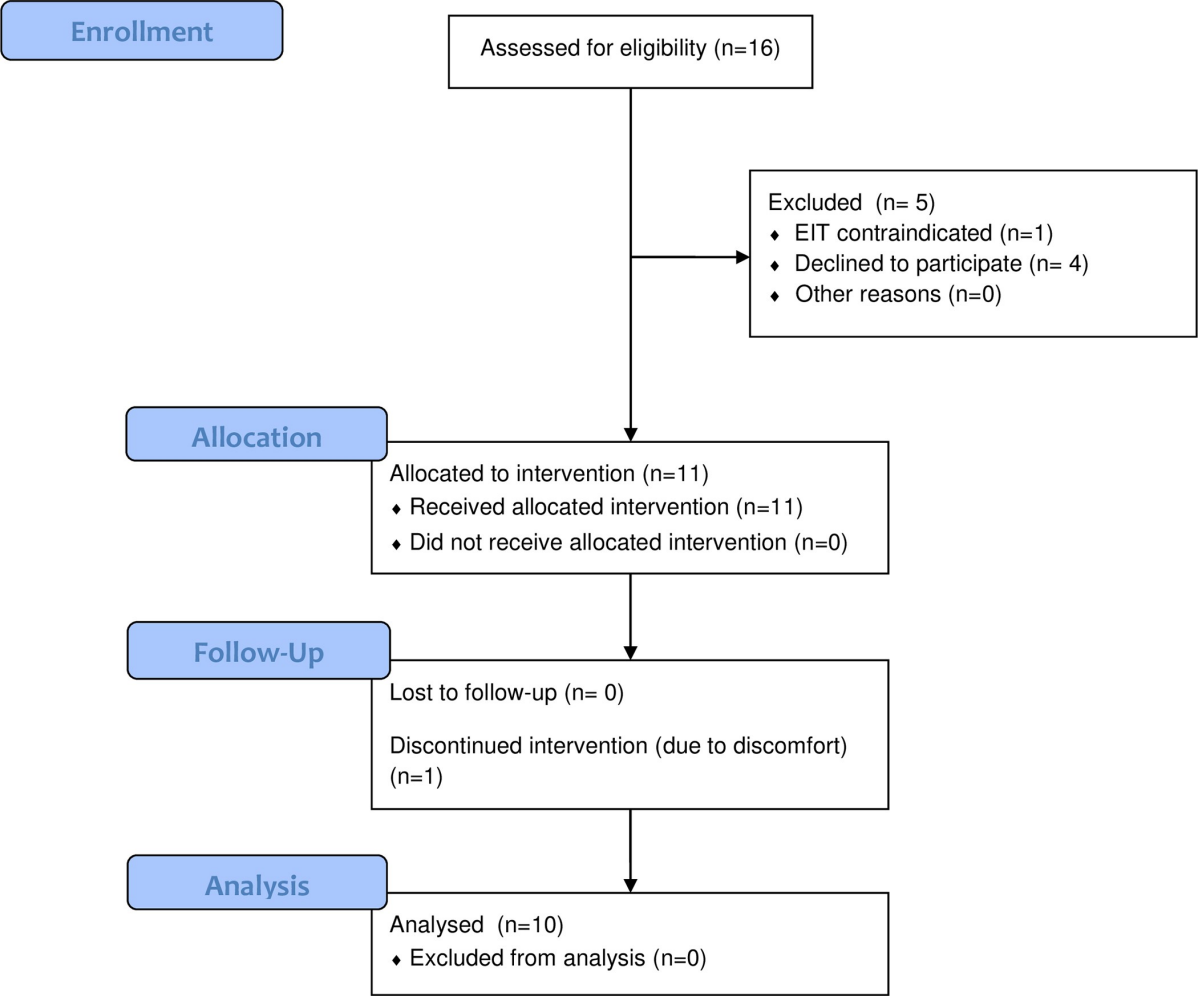
CONSORT flow chart of included patients. The flow chart was modified to the non-randomized design of the current investigation. EIT, electrical impedance tomography.

From January 4 2021 to May 23 2021, eleven patients were included in the study (Fig 1). One patient aborted the study after 20 minutes in the prone position due to back pain, leaving ten patients for the final analyses. Participant characteristics are summarized in Table 1. All participants were male with a median (IQR) age of 64 (44–73) years and a body mass index of 26 (25–29) kg m^-2^. Half of the patients had HFNO, and half had NIV for respiratory support. Median (IQR) PaO_2_/FiO_2_ ratio at inclusion was 18.9 (14.7–21.5) kPa (142 (110–161) mmHg) in the supine position. Hypertension and diabetes mellitus were the most common comorbidities. All patients received thromboprophylaxis with low-molecular weight heparin and were treated with dexamethasone. Six (60%) patients had ongoing continuous intravenous sedation with dexmedetomidine.

**Table 1:** Patient characteristics of the study cohort.

Patient characteristics	All patients (n = 10)
Age (years)	64 (47–73)
Sex (male)	10 (100%)
Weight (kg)	85 (78–97)
Height (cm)	178 (173–185)
BMI (kg m^-2^)	26 (25–29)
Obesity (BMI >30 kg m^-2^)	2 (20%)
Hypertension	4 (40%)
Diabetes mellitus	4 (40%)
Heart disease	0 (0%)
Previous or current PE	0 (0%)
Asthma	2 (20%)
COPD	0 (0%)
Chronic renal failure	1 (10%)
Malignancy	1 (10%)
Active smoker	0 (0%)
Previous smoker	4 (40%)
Days with symptoms	13 (9–16)
Days hospitalized	5 (4–9)
Days ICU	3 (2–6)
HFNO	5 (50%)
Flow (L min^-1^) median (range)	50 (50–60)
NIV	5 (50%)
PEEP (cmH_2_O) median (range)	8 (5–12)
PS (cmH_2_O) median (range)	5 (3–5)
FiO_2_	0.5 (0.4–0.65)
SpO_2_ (%)	94 (93–95)
PaO_2_ (kPa)	9.6 (8.9–10.0)
PaO_2_ (mmHg)	72 (67–75)
PaO_2_/FiO_2_ ratio (kPa)	18.9 (14.7–21.5)
PaO_2_/FiO_2_ ratio (mmHg)	142 (110–161)
Heart rate (min^-1^)	66 (55–75)
Mean arterial pressure (mmHg)	80 (72–86)
Remdesevir	4 (40%)
LMWH prophylaxis	10 (100%)
Dexamethasone	10 (100%)
Antibiotics	5 (50%)
Vasopressor	0 (0%)
Dexmedetomidine	6 (60%)
C-reactive protein (mg L^-1^)	54 (21–66)
Procalcitonin (μg L^-1^)	0.19 (0.11–0.60)
Ferritin (n = 9) (μg L^-1^)	985 (848–1348)
Hemoglobin (g L^-1^)	141 (129–148)
D-dimer (mg L^-1^)	1.2 (0.5–2.1)
Creatinine (μmol L^-1^)	71 (26–110)
Troponin-I (ng L^-1^)	9 (6–13)
NT-pro-BNP (n = 9) (ng L^-1^)	243 (157–494)
SOFA score	3 (3–5)
Temperature (°C)	37.1 (36.7–37.8)

Data are presented as median (IQR) or numbers (percentages). BMI, body mass index. HFNO, high-flow nasal oxygen. NIV, non-invasive ventilation. PEEP, positive end-expiratory pressure. PS, pressure support. COPD, chronic obstructive pulmonary disease. LMWH, low molecular weight heparin. NT-pro-BNP, N-terminal prohormone of brain natriuretic peptide. PE, Pulmonary embolism. SOFA, sequential organ failure assessment.

### Primary outcome variables–electrical impedance tomography

#### Global and regional tidal impedance variation

**Fig 2:**
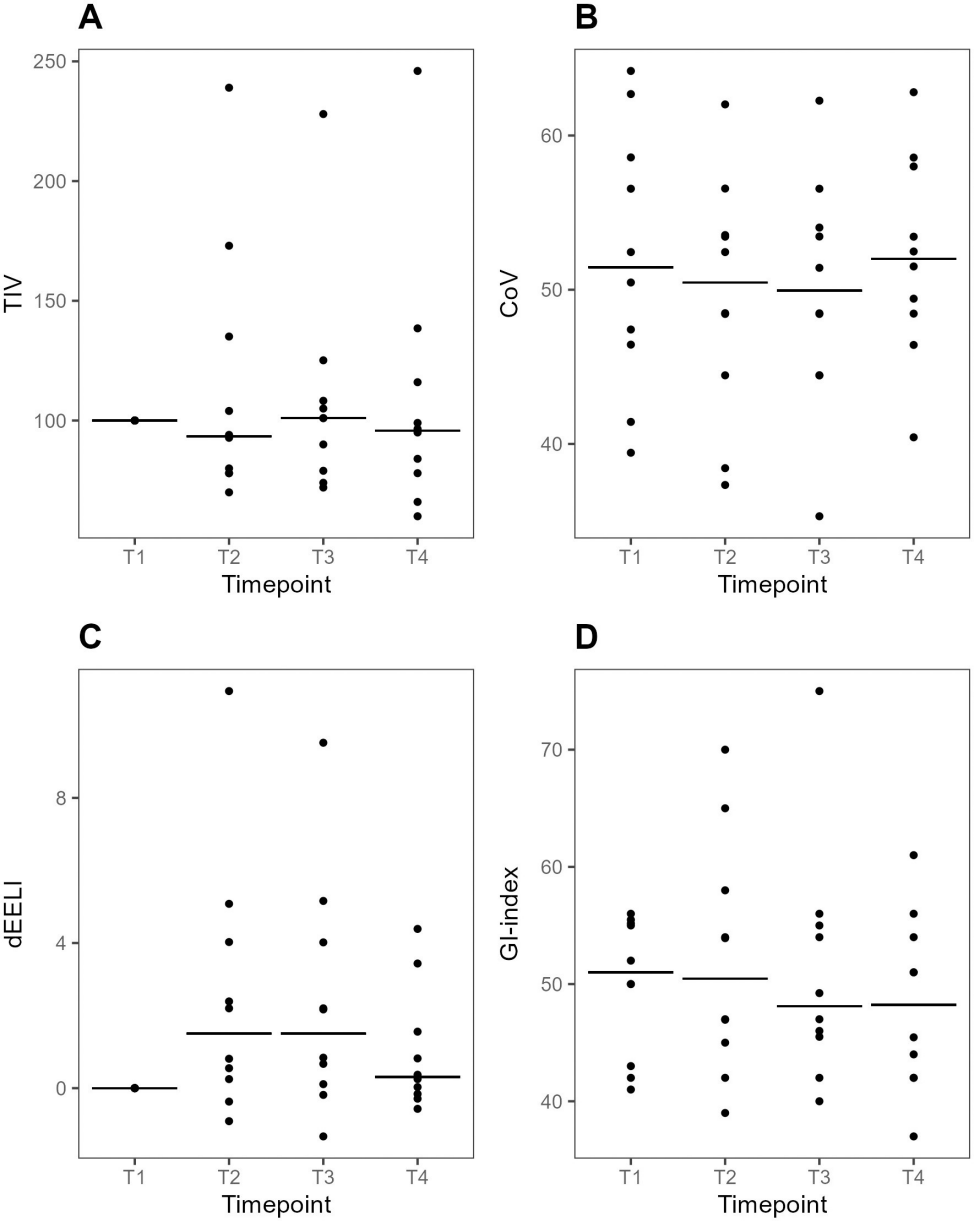
Plots of global EIT-variables at supine baseline, during prone positioning and after supine repositioning. Individual values and medians at each time point. A. Tidal impedance variation (TIV) relative to T1 (reference value 100 AU). B. Center of ventilation (CoV). At CoV = 50%, the gravitation point of the ventilation is at the center of the chest diameter. A higher number indicates a ventral over-weight whereas a lower number indicates a dorsal over-weight of the ventilation distribution. C. Delta end-expiratory lung impedance (dEELI) relative to T1 (reference value 0). D. Global inhomogeneity (GI) index. A higher value indicates a more heterogenous spatial distribution of ventilation across the lung. dEELI at T3 was higher than dEELI at T1, median difference 1.51 (95% CI 0.19–5.16, p = 0.04). T1-4: predefined data collection timepoints. T1, Supine baseline. T2, 30 min after prone positioning. T3, 60 min after prone positioning. T4, 30 min after supine repositioning.

TIV did not change between T3 and T1 (Fig 2). Analyzing individual responses four (40%) patients showed a reduction (>10%) of TIV_T3_ compared with TIV_T1_ and two (20%) patients showed an increase (>10%). At T4 four patients had an increase in TIV and three patients showed a decrease.

**Fig 3:**
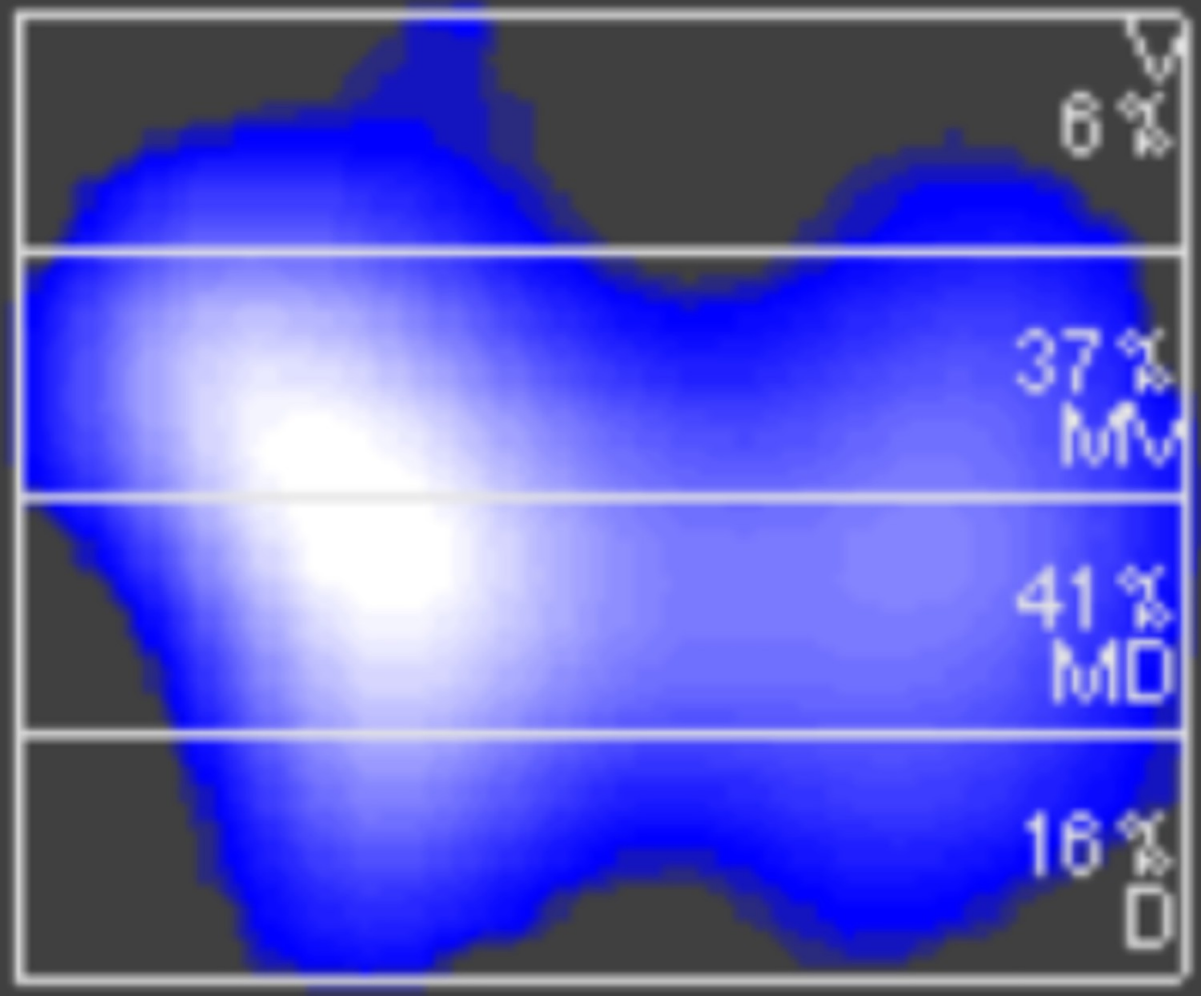
Example of a reconstructed EIT-image horizontally divided into four equally spaced regions of interests. Percentages in each region of interest represent the fraction of global tidal impedance variation. EIT, electrical impedance tomography. D, Dorsal. MD, mid-dorsal. MV, mid-ventral. V, ventral.

**Fig 4:**
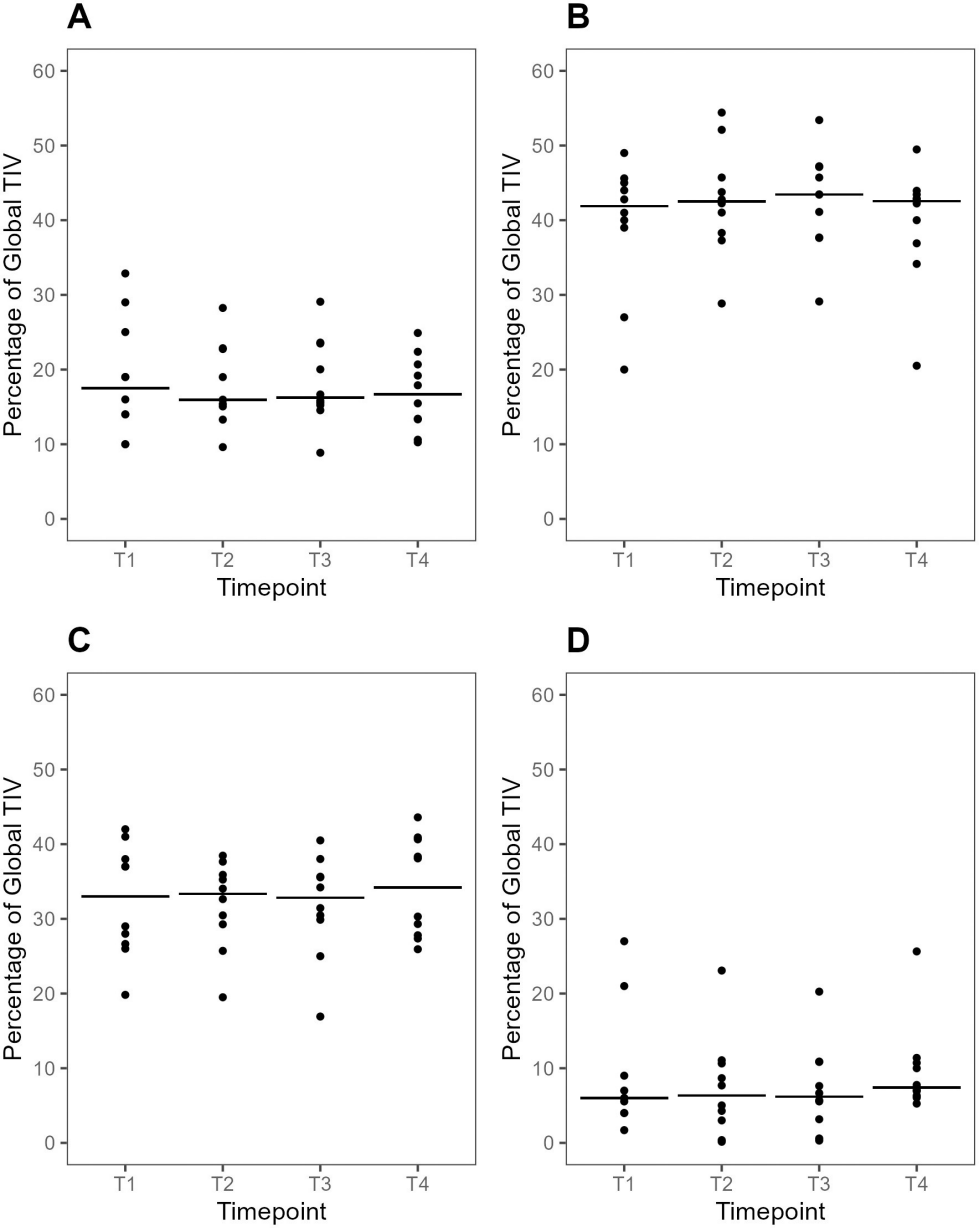
Plots of TIV-distribution per dorsal (A), mid-dorsal (B), mid-ventral (C) and ventral (D) ROI at supine baseline, during prone positioning and after supine repositioning. Individual values and medians at each time point. TIV is expressed as the fraction of global TIV (%). There were no significant differences between T3 and T1 in any of the ROIs (p >0.05 for all comparisons). TIV, tidal impedance variation. ROI, region of interest. T1-4: predefined data collection timepoints. T1, Supine baseline. T2, 30 min after prone positioning. T3, 60 min after prone positioning. T4, 30 min after supine repositioning.

In all participants and regardless of body position, most of the TIV was distributed to the mid-ventral and mid-dorsal ROI (Fig 3), together contributing with a median (IQR) 72 (68–77)% of global TIV_T1_ at baseline (Fig 4). Ventilation distribution within ROIs were similar at all timepoints (Fig 4).

#### Center of ventilation

CoV did not change between T3 and T1 APP (Fig 2). At an arbitrary threshold of 10% relative change compared with CoV_T1_, four patients (40%) had a dorsal shift of ventilation at T3, all reversed at T4. Four patients (40%) had no shift of ventilation at T3. Two patients (20%) had a ventral shift of ventilation at T3, of whom one patient (10%) had persistent ventral shift.

#### Global and regional delta end-expiratory lung impedance

dEELI_T3_ was higher compared with dEELI_T1_, median difference 1.51 (95% CI 0.19–5.16, p = 0.04), (Fig 2). Using an arbitrary threshold of 0.20 change at T3 compared with T1, seven (70%) patients showed an increase in dEELI. One (10%) patient showed a decrease, and two (20%) patients had no change in in dEELI_T3_, compared with dEELI_T1_. Three (30%) patients had a persistent increase in dEELI at T4 compared to T1. In all other cases, changes during APP were reversed to near baseline values at dEELI_T4_.

**Fig 5:**
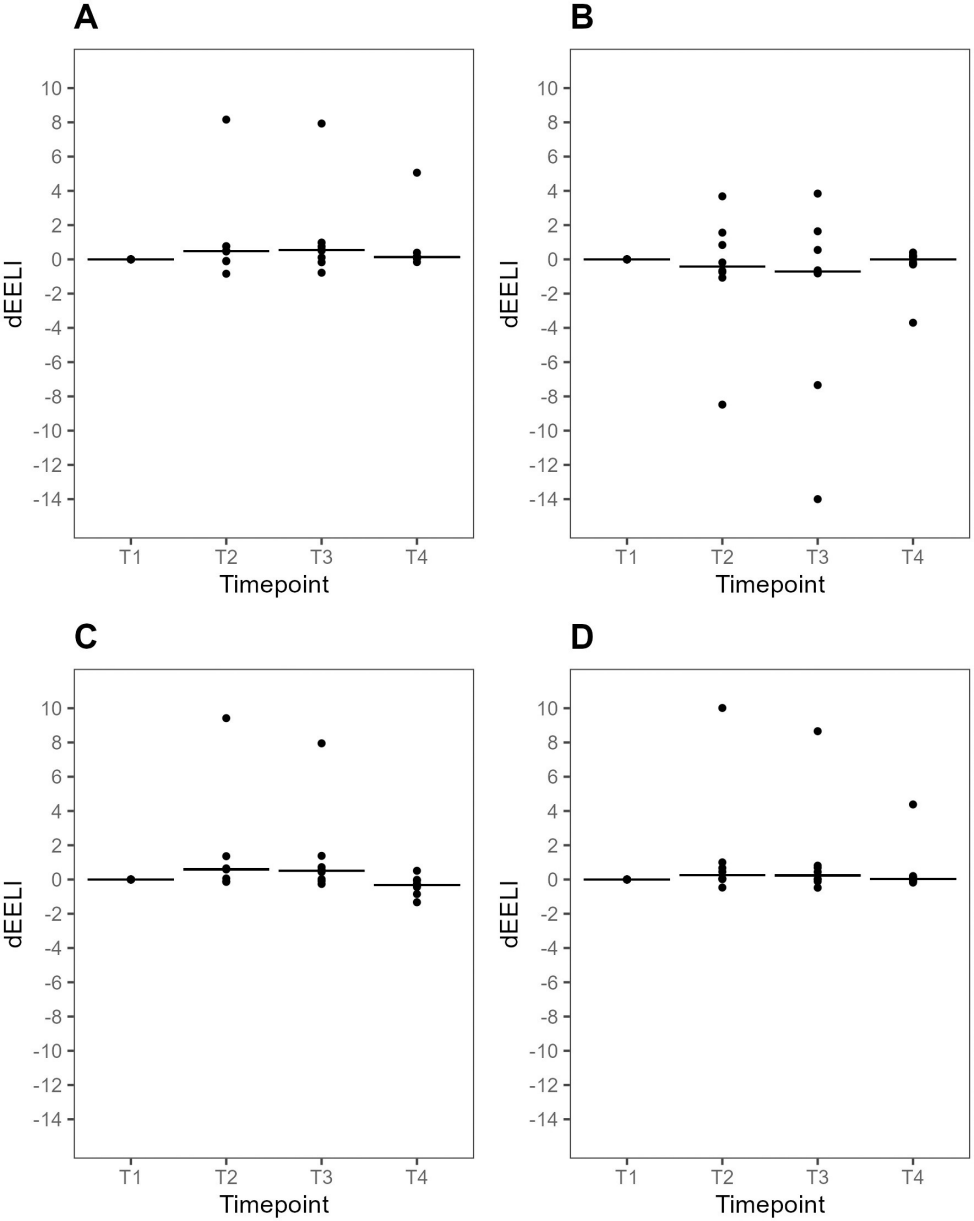
Plots of dEELI per dorsal (A), mid-dorsal (B), mid-ventral (C) and ventral (D) ROI at supine baseline, during prone positioning and after supine repositioning. Individual values and medians at each time point. Data were available for eight (80%) patients. There were no significant differences between T3 and T1 in any of the ROIs (p >0.05 for all comparisons). dEELI, delta end-expiratory lung impedance relative to T1 (reference value 0). T1-4: predefined data collection timepoints. T1, Supine baseline. T2, 30 min after prone positioning. T3, 60 min after prone positioning. T4, 30 min after supine repositioning.

Analyses of regional dEELI were available for eight patients. There was a reduction of dEELI in the mid-dorsal ROI and an increase in the three other ROIs at T3 compared with T1, although these changes were not statistically significant (Fig 5).

#### Global inhomogeneity index

GI-index values were similar at T3 and T1 (Fig 2). At an arbitrary cut-off of 10% change at T3 compared with T1, two (20%) patients had an increase, three (30%) patients had a decrease, and five 50%) patients had no change at T3 compared with T1. Two patients had a change of GI-index_T4_ compared with GI-index_T1_ of whom one (10%) patient had an increase, and one (10%) patient had a decrease.

All results are also presented in S1 Table.

#### Secondary outcome variables

**Fig 6:**
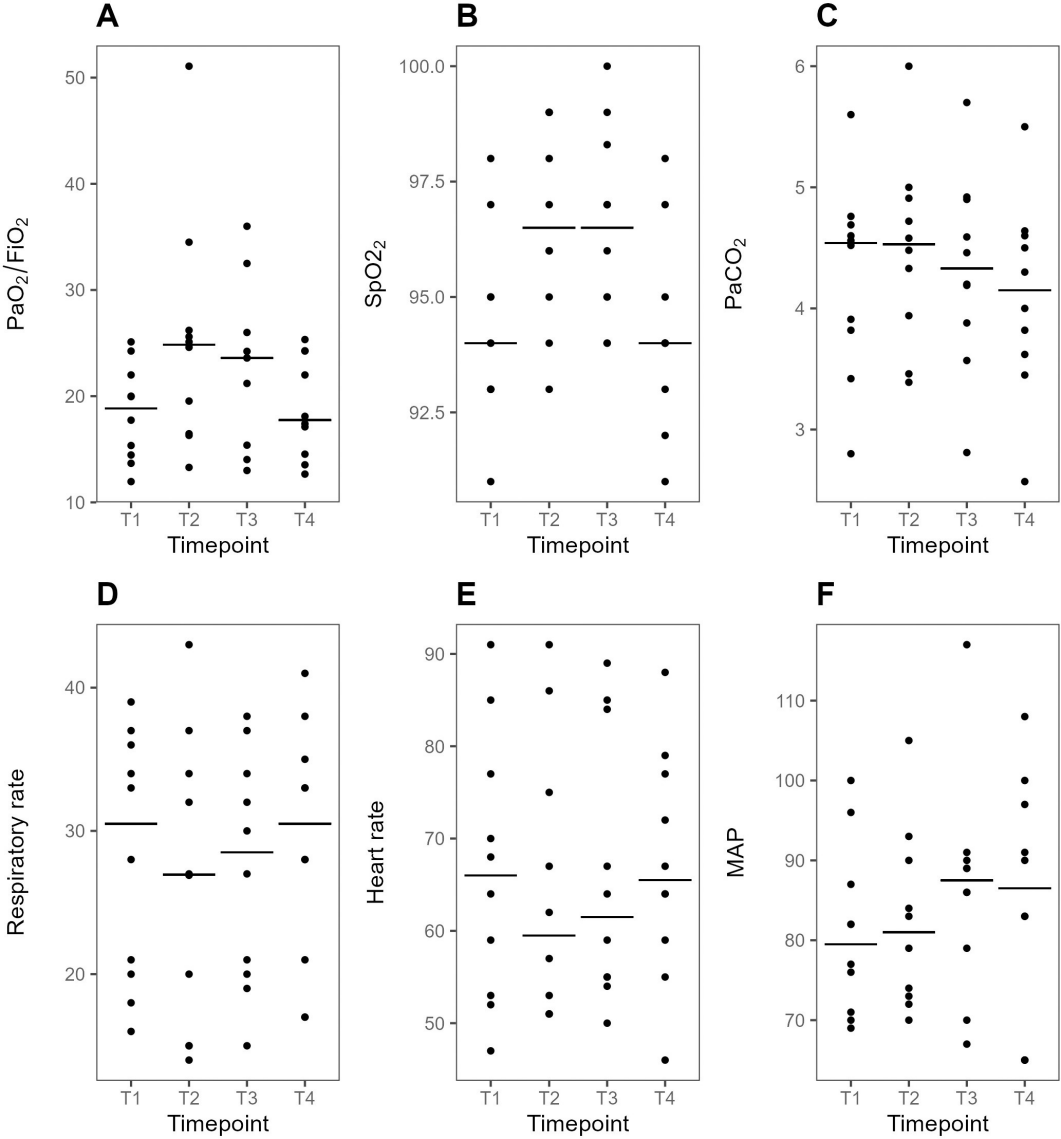
Plots of secondary outcome variables at supine baseline, during prone positioning and after supine repositioning. Individual values and medians at each time point. A, FiO_2_/PaO_2_ ratio (kPa). B, SpO_2_ (%). C PaCO_2_ (kPa). D, Respiratory rate (RR min^-1^) E, Heart Rate (HR min^-1^). F, Mean arterial pressure (MAP, mmHg). PaO2/FiO2 ratio (median difference 2.9 kPa, 95% CI 0.9–8.3) and SpO2 (median difference 3% (95%CI 0–4) were higher at T3 compared with T1 but there were otherwise not significant differences between T3 and T1 for the other presented variables. T1-4: predefined data collection timepoints. T1, Supine baseline. T2, 30 min after prone positioning. T3, 60 min after prone positioning. T4, 30 min after supine repositioning.

PaO_2_/FiO_2_ ratio and SpO_2_ transiently increased at T3 compared to T1 (Fig 6). Seven (70%) patients responded with >10% increase in PaO_2_/FiO_2_ ratio at T3 compared with T1 of whom 3 (30%) had >20% increase. Two (20%) of these patients had a persistent increase at T4. Respiratory rate heart, PaCO_2_ and hemodynamical variables were similar between T3 and T1 (p>0.05 for all comparisons). There was a negative correlation between the change of dEELI and PaO2/FiO2 ratio from T1 to T3 (rho = -0.66, p = 0.04) (S1 Fig).

Three (30%) patients showed a combination of reduced TIV and reduced or unaltered RR at T3 compared with T1. These patients also had an increase in PaO_2_/FiO_2_ ratio, an increase in dEELI and reduced or unaltered GI-index.

#### Adverse effects

One patient was excluded from the study because of inability to complete the protocol secondary to discomfort. In the ten patients who completed the study protocol, no adverse events were observed.

## Discussion

There were several key findings in this study. First, 70% of the patients showed an increase in global dEELI during APP. Second, APP had no effect on ventilation distribution or ventilation homogeneity. Third, oxygenation improved in most patients during APP. Fourth, changes in EIT-variables during APP returned to near baseline values after supine repositioning in most patients.

The observed improvement in dEELI corroborates the findings in a recently published study [17] and suggests that lung recruitment is an important physiological effect of APP in patients with severe COVID-19 pneumonia. However, although most patients also responded to APP with improved oxygenation in line with previous reports [34] oxygenation changes between the supine and the prone position were inversely correlated with changes in dEELI. This correlation should be interpreted with caution as this was a post hoc analysis of our study requiring confirmation in larger cohorts. In contrast to our result, Fossali et al. found no correlation between lung recruitment and oxygenation in their cohort of 21 COVID-19 patients although they studied intubated patients [35]. However, both our results and the results by Fossali indicate that improved oxygenation during prone position does not equate to lung recruitment. An increase in oxygenation following prone positioning may be due to changes in ventilation (V), perfusion (Q) or both if it results in improved V/Q-matching [36, 37]. In invasively ventilated patients, the main benefit of prone positioning is however likely due to improvements in respiratory mechanics allowing for lung-protective ventilation [11] rather than increases in oxygenation due to improved V/Q matching. In support of this, changes in PaO_2_/FiO_2_ ratio during prone positioning did not predict survival in intubated patients with moderate to severe ARDS in a post-hoc analysis of the landmark PROSEVA study [9]. Patients had benefit regardless of changes in oxygenation after prone positioning. Similarly, an increase in PaO_2_/FiO_2_ ratio during APP was not associated with a reduction of intubation rates in a randomized controlled trial of COVID-19 patients [10]. In contrast to indices of oxygenation, EIT potentially identifies patients with favorable alterations of respiratory mechanics, as well as those with possible harmful effects. EIT may thus improve the selection of patients that benefits from APP compared with oxygenation measurements.

Long duration of prone positioning is crucial to achieve mortality reduction in invasively intubated patients with ARDS [38] and longer duration of APP is likewise associated with a lower risk of intubation and death in patients with severe COVID-19 [5, 7, 10]. Alveolar recruitment during APP may improve respiratory compliance, reduce work or breathing [39] and attenuate the possible harmful effects of vigorous spontaneous breathing efforts in patients with respiratory failure [40]. The reversal of dEELI that occurred after supine repositioning in most patients in the current investigation suggests that lung recruitment is confined to the period of APP only, providing a possible mechanism that may explain some of the dose-dependent effects of APP in COVID-19 patients.

Although dorsal redistribution of ventilation is common during prone positioning among intubated patients [14, 35], there were no changes in ventilation distribution during APP as assessed by regional TIV-distribution and CoV. Similar to our findings, APP did not cause shifts in regional TIV during APP in a previous study of sixteen COVID-19 patients [14]. However, contrary to our results, Grieco et al. reported a shift of regional TIV to dependent lung regions during APP [17]. The differences in our results may have several explanations: 1) We used a 10-degree trunk inclination for both the supine and the prone position whereas Grieco et al. used the semi recumbent position compared to the prone position. The difference in trunk inclination between the semi recumbent and prone position in their study may have confounded the results with effects of trunk inclination on lung mechanics [41–43]. 2) The duration of APP was shorter in our study (60 min vs 120 min) and some effects of APP may take longer time to establish; 3) Grieco et al. investigated APP in patients with respiratory failure of all causes whereas we investigated patients with COVID-19 only. The lack of effect on ventilation distribution in our study was further supported by the GI-index which did not change during or after APP. This finding is corroborated by a previous study by Brunelle et al. which reported no change in GI-index or CoV between the supine and prone position in patients with COVID-19. Patients in their study had less advanced respiratory support (80% HFNO, 20% non-rebreather) compared with our investigation where 50% had HFNO and NIV respectively. Our results hence extend this findings to patients with more advanced degree of respiratory failure [16]. In the present study, GI-index was about 25% higher at baseline than in healthy controls in previous studies and comparable to findings in both spontaneously breathing supine COVID-19 patients as well as intubated ARDS-patients [15, 33]. Hence, COVID-19 pneumonia likely causes significant ventilation heterogeneity. The clinical significance of this finding, specifically, the effects on long term outcomes associated with changes of the GI-index in response to therapeutic interventions are unknown.

In this cohort, a subgroup of three patients responded to APP with a combination of a reduction of TIV and RR, an increase in oxygenation and dEELI and an unaltered or decreased GI-index. This suggests a reduction of the work of breathing in combination with lung recruitment, improvement in oxygenation and unaltered or decreased ventilation heterogeneity. These patients may represent a subgroup of patients with particularly favorable response to APP that may be further investigated in future studies.

There are several strengths of this study. First, The degree of respiratory failure, preexisting comorbidities and patient characteristics were similar to those described in previous trials of APP in COVID-19 patients increasing external validity [7]. Second, we did not change the head up inclination of the bed between the supine and the prone position, although the 30-degree semi recumbent position could be argued to be more clinically relevant when patients are positioned on their backs. There is an increasing amount of ICU research reporting that the degree of trunk inclination ranging from the supine to the semi recumbent position have significant impact on lung mechanics and end-expiratory lung volume [41–43]. Thus, by leaving the bed inclination unchanged throughout the study protocol, we excluded these confounders and isolated the effects of prone positioning on lung aeration only. Moreover, if the bed position is altered between the supine and prone position, changes in the position of the diaphragm may alter the section of the lung that is being detected by EIT. Hence, comparing the semi recumbent position at 30 degrees to the prone position at 10 degrees would increase the risk of detecting changes between different sections of the lung rather than changes imposed by positional change in the same lung section. However, this is also a limitation of our results as the 30-degree head up position is standard care. Third, this comprehensive report provides novel insights in lung physiology alterations induced by APP in COVID-19 patients with hypoxemic respiratory failure and suggests new ways to select patients for APP that may guide future research.

There are also several limitations to consider in the interpretation of the study results. First, sample size was small, and imprecision precluded precise estimation of effects and differences and investigation of potentially important subgroups. Specifically, patients included with HFNO and NIV respectively may have different lung physiological response to APP and should be targeted separately in future research. Second, this was an exploratory observational study with no *a priori* power calculation or predefined single primary outcome and results should therefore be considered hypothesis generating only. Third, all included patients were men and it is uncertain whether the results are generalizable to women. Fourth, we only studied changes in lung aeration. Assessment of simultaneous lung perfusion would have produced data on V/Q matching increasing the understanding of the impact of APP on pulmonary shunt and global oxygenation. However, the equipment used in this study did not allow for these measurements. Fifth, as patient tolerance to APP may be low, APP duration in this study was relatively short to maximize the number of patients able to complete the protocol. Thus, we cannot exclude the possibility that changes in EIT-variables may evolve over longer periods of time, although changes after 30 and 60 minutes of APP were similar. Finally, we investigated short term physiological effects only. Association of EIT-variables during APP with long-term outcomes remains to be further investigated.

## Conclusions

In this small cohort of COVID-19 patients, awake prone positioning was associated with changes consistent with a transient lung recruitment without important changes in ventilation distribution. If these results are confirmed in larger cohorts, future research is warranted to determine whether EIT-guided selection of APP-candidates is associated with improved long-term outcomes.

## Supporting information

S1 Checklist The Transparent Reporting of Evaluations with Nonrandomized Designs (TREND) checklist. (PDF)

S1 File Study protocol. (PDF)

S1 Table Outcome variables of the study cohort at supine baseline, during prone positioning and after supine repositioning. (DOCX)

S1 Fig Correlation analysis between change of PaO2/FiO2 and dEELI from supine to prone position. (PDF)
