# Supplementary material for: Lung impedance changes during awake prone positioning in COVID-19. A non-randomized cross-over study
Source: PLoS One. 2024 Feb 21;19(2):e0299199. doi: 10.1371/journal.pone.0299199 (PMC10880988; doi:10.1371/journal.pone.0299199)
Supplement: S1 File — (PDF) [file pone.0299199.s002.pdf]

TRIAL PROTOCOL

Awake prone positioning with high flow nasal oxygen in critically ill covid-19 patients

The PROFLO Trial

Swedish title

Buklägesbehandling med högflödesgrimma vid covid-19.

Principal investigator

Associate professor Peter Frykholm

CONTENTS

PROTOCOL SYNOPSIS

GLOSSARY OF ABBREVIATIONS

1. STUDY MANAGEMENT

2. INTRODUCTION AND BACKGROUND

3. STUDY DESIGN

4. PARTICIPANTS AND RECRUITMENT

5. INTERVENTION

6. RANDOMISATION AND BLINDING

7. OUTCOMES

8. STATISTICAL METHODS

9. ADVERSE EVENTS

10.PRONE META-TRIAL

11. REFERENCES

PROTOCOL SYNOPSIS

English title: Awake prone positioning with high flow nasal oxygen in critically ill covid-19 patients.
Swedish title: Buklägesbehandling med högflödesgrimma vid covid-19.
Objectives: Determine if a protocol for prone positioning with high flow nasal cannula or noninvasive ventilation reduces the rate of intubation in awake spontaneous breathing covid-19 patients or patients with other infectious disease with moderate to severe hypoxemic respiratory failure.
Design: Multicenter randomized controlled, parallel group, superiority trial.

Population and Intervention: 240 adults, 18 years or older with confirmed or strongly suspected infection with novel corona virus SARS-CoV-2 or other infectious disease, ongoing or planned oxygen treatment with high flow nasal cannula or noninvasive ventilation and moderate to severe respiratory failure as defined by a PaO_2/FiO2 ratio of ≤ 20 kPa (=150 mmHg) and/or a FiO_2 of 0.5 required to reach an SpO2 of 94%.
Patients will be randomized 1:1 to a prone positioning protocol with a prone position target of 16 hours/day OR standard care.
Outcomes: Primary outcome is the rate of intubation. Secondary outcomes include time in prone position, admission to the ICU, time on mechanical ventilation, need for renal replacement therapy, the WHO ordinal scale for clinical improvement and the 7- and 30-day mortality and complication rate.

GLOSSARY OF ABBREVIATIONS

ARDS Acute respiratory distress syndrome

COVID-19 Corona virus disease 2019

EELI End expiratory lung impedance

EELV End expiratory lung volume

EIT Electrical impedance tomography

FiO_2 Fraction of inspired oxygen

HFNC High flow nasal cannula

GCS Glasgow Coma Scale

ICU Intensive care unit

LOS Length of stay

NIV Non-invasive ventilation

PP Prone position/-ing

PaO_2 Partial pressure of arterial oxygen

RLS Reaction Level Scale

SpO_2 Peripheral capillary oxyhaemoglobin saturation

TIV Tidal impedance variation

VAS Visual analogue scale

1. Study management

1.1 Principal investigator

Associate professor Peter Frykholm

Department of Surgical Sciences, section of Anaesthesia and Intensive Care Medicine, Uppsala university, Sweden.

1.2 Trial Steering Commitee

Peter Frykholm, Uppsala University Hospital, Uppsala Sweden.

Jacob Rosén, Uppsala University Hospital, Uppsala, Sweden.

Diddi Fors, Uppsala University Hospital, Uppsala, Sweden.

Malin Jonsson-Fagerlund, Karolinska University Hospital, Uppsala Sweden

Erik von Oelreich, Karolinska Hospital, Solna, Sweden. erik.von.oelreich@gmail.com

Knut Taxbro, Dept of Anaesthesia and Intensive Care, Jönköping Hospital, Jönköping, Sweden. knut.taxbro@rjl.se

1.3 Data Monitoring Committee

Professor Miklos Lipcsey, Dept of Surgical Sciences, Uppsala University, Uppsala, Sweden. Miklos.lipcsey@surgsci.uu.se

Professor Emeritus Anders Larsson, Dept of Surgical Sciences, Uppsala University, Uppsala, Sweden. Anders.Larsson@surgsci.uu.se

Nermin Hadziosmanovic, Uppsala Clinical Research Centre, Uppsala, Sweden. nermin.hadziosmanovic@ucr.uu.se

2. INTRODUCTION

The COVID-19 pandemic has been rapidly expanding since the first reported case in Wuhan, China in December 2019, now affecting almost all the world's countries/territories/areas with more than 3.000.000 confirmed cases and more 207.973 deaths as of April 29[1]. COVID-19 can lead to pneumonia with severe acute hypoxemic respiratory failure with the need for high FiO_2 and invasive ventilation[2].

HFNC supplies heated humidified oxygen through the nose and has become a mainstay in the treatment of acute hypoxemic respiratory failure in ICUs, reducing the risk of intubation and mechanical ventilation[3-5]. HFNC increases airway pressure and lung volume directly related to the flow and inversely related to mouth opening and reduces the work of breathing[6-10].

The use of PP in ARDS increases the PaO_2/FiO_2-ratio and reduces mortality in moderate to severe ARDS[11-14]. PP in non-intubated spontaneously patients- awake PP- is feasible and transiently improves oxygenation in patients with hypoxemic acute respiratory failure[15]. Further, awake PP may decrease intubation rates in patients with moderate ARDS[16].

Facing an overwhelming inflow of patients with COVID-19 associated severe respiratory syndrome, ventilator shortage has already become a reality in some centers. Further, invasive mechanical ventilation is associated with a high mortality rate[17,18]. Therefore, strategies to reduce the risk of intubation and mechanical ventilation are needed.

2.1 Aim(s)

The primary aim of this study is to determine the effect on prone positioning with HFNC or NIV on the intubation rate in critically ill COVID-19 patients or patients with other infectious disease with moderate to severe hypoxemic respiratory failure. The secondary aims include time/day in PP, 7- and 30-day mortality, days on ventilator, inhospital and ICU LOS, incidence of RRT, the need for vasopressors and/or inotropic drugs, the WHO ordinal scale for clinical improvement, and adverse events during PP. In a subset of 30 patients we aim to study the acute effects on lung volume and function with EIT, PaO2/FiO2 ratios, respiratory rate, hemodynamic parameters and effect on dyspnea.

2.2 Meaning

This study will determine the effect of awake PP with HFNC or NIV on the rate of intubation in severe hypoxemic respiratory failure due to COVID-19 pneumonia or other infectious diseases. The combination of HFNC/NIV and awake PP may be valuable in situations where ventilator resources are overwhelmed by patients in need of invasive mechanical ventilation support. This study will further contribute to the understanding of physiological mechanisms responsible for improved oxygenation previously observed in PP by examining changes in global and regional lung function in a subset of patients.

3. STUDY DESIGN

3.1 Study type

This is a multicenter open labelled randomized controlled, parallel group, assessor blind superiority trial.

3.2 Ethical considerations

Ethical approval will be sought from the Swedish Ethical Review Authority. Written informed consent will be obtained from all participants in the study. This study will be conducted in compliance with all stipulations of this protocol, the conditions of the ethics committee approval, standards of Good Clinical Practice (as defined by the International Conference on Harmonisation), ethical principles that have their origin in the Declaration of Helsinki and all applicable national and local regulations. This study is not sponsored by any commercial entity.

3.2 Study setting

The intervention will be performed in covid-19 cohort patient wards of infectious disease departments and intensive care units of major hospitals in Sweden.

3.3 Study registration

The study is registered at ISRCTN (https://doi.org/10.1186/ISRCTN54917435).

4. PARTICIPANTS AND RECRUITMENT

4.1 Number of participants

240 male or female adults 18 years or older will be enrolled.

4.2 Eligibility criteria

Patients will be randomized in the study only if they meet all of the inclusion criteria and none of the exclusion criteria.

4.2.1 Inclusion criteria

Each patient must meet all the inclusion criteria to be enrolled in this study:

- Age 18 year or older
- Admitted to the hospital with confirmed or strongly suspected (e.g due to history, symptoms, radiology, laboratory tests) COVID-19 infection or other infectious disease.
- Moderate to severe hypoxemic respiratory failure defined as a PaO_2/FiO_2 ratio ≤ 20 kPa (or 150 if mmHg is used) and/or a FiO2 of ≥ 0.5 to reach a SpO_2 of 94%
- Oxygen supplementation (ongoing or planned) with HFNC or NIV.

4.2.2 Exclusion criteria

- Severe nasal obstruction or contraindication to HFNC or NIV.
- Patient unable to lay prone or in the face forward position (e.g. due to morbid obesity, abdominal wounds etc).
- Immediate need for intubation.
- Severe and/or uncontrolled hemodynamic instability.
- Previous intubation for COVID-19 pneumonia (i.e. step-down patients).
- Pregnancy.
- Known terminal illness with life expectancy less than 1 yr.
- Decision not to intubate.
- Inability to understand instructions and/or to cooperate with instructions necessary to complete the allocated intervention.
- Inability to understand oral or written study information.
- GCS less than 14 or RLS less than 2.

4.3 Recruitment, identification and consent of potential participants

Potential participants will be identified as they change oxygen therapy from standard nasal cannula or mask to high flow nasal cannula or NIV. When a FiO2 of 0.5 is required to reach a SpO2 of 94% the patients will be approached.
Patients will receive oral, followed by written information and provided an appropriate time for consideration and if needed, consultation with the treating physician, a family member, and/or another person of their choice. Written informed consent will be obtained prior to recruitment into the trial. Due to restrictions in transfer of e.g. paper from single patient rooms, the informed consent procedure may include a proxy in the room transferring the information to and from the patient, or a digital transfer (photo of the informed consent form)

4.4 Subject withdrawal

Any refusal or withdrawals from the study will be documented, with the reason recorded. The investigator or treating physician may withdraw a patient from the study at any time if it is deemed it is no longer safe to continue with the allocated treatment.

5. INTERVENTIONS

5.1 Treatment arms

There are two arms, the prone positioning arm and the standard care arm.

5.1.2 Standard care arm

PP is neither prohibited nor encouraged and may be prescribed by the treating physician at their discretion.

5.1.2 Prone positioning arm

The patient, the treating physician/-s and other providers of care are informed of the study allocation and a protocol for PP is initiated with a target PP of 16/24 hours. Prone positioning is defined as:

1. Prone position. Pillows may be used. Patient may position their arms at their choice. Bed may be at zero degrees or in a reverse Trendelenburg position as comfortable.
2. Left or right face forward position. This is to increase comfort and compliance to the protocol.

PP should be performed in coherent periods of at least two hours at a time but may be individualized (i.e. longer or shorter) as required to maximise compliance and/or to reach the target time. To be able to reach the target of 16/24 hours, PP should ideally be performed during sleep. When not in PP but in bed, patients should lay in the semi-recumbent position with the head of the bed elevated to 30 degrees or the left or right recumbent position. Flat supine positioning is discouraged. The time spent in other positions than PP should be planned for meals etc. In the ICU, sedation is allowed as indicated but is not protocolized.

5.3 Other aspect of care for both arms

All other interventions will follow local guidelines at the present hospital and are not affected by the study protocol. A change of oxygen therapy from HFNC to NIV or vice versa is allowed in both groups at the treating physicians discretion, however the protocol for PP is continued. If patients are transported in-hospital, oxygenation by face mask is allowed and the patient can be transported in a position appropriate for adequate monitoring and safety. The decision to intubate is made at the discretion of the treating intensivist. Patient positioning after intubation is not protocolized.

5.4 Protocol termination

Every participant follows the allocated intervention until protocol termination criteria are met. Termination criteria are fulfilled if:

- the patient recovers and standard nasal prongs or open face mask (”oxymask”) with a maximal flow of 5 L/min can be used to manage the hypoxemia for at least 12 hours.
- the patient is intubated.

- death occurs.

5.5 Electrical impedance tomography - subgroup study

EIT is a non-invasive radiation-free imaging technique that provides real-time images and data of regional lung ventilation and lung volumes[19]. Changes in EELI and TIV correlates with EELV and tidal volume respectively.

5.5.2 Participants, identification and recruitment

60 patients (15 patients allocated to PP and 15 patients allocated to standard care in the groups of patients treated with HFNC and NIV respectively) that are admitted to the ICU and previously included in the present trial. Participants will have signed a written informed consent for this part of the study at inclusion but may withdraw from this subgroup study when approached at the ICU or at any other time.
Amendment February 2021: if inclusion of patients for the main study is terminated prematurely after an interim analysis due to futility or efficacy, we plan to continue inclusion of patients only for the physiologic study the effects of prone position on lung aeration according to 5.5.1.

5.5.1 EIT protocol and measurements.

An elastic EIT-band will be placed around the lower thoracic wall and connected to an EIT-device. Participants will be put in a semi-recumbent position in the bed, with the head elevated to 30 degrees and receive HFNC at a rate of 50 L/min or NIV with the current individual settings, including the FiO_2 required to reach an SpO_2 of 92-96%. After stable SpO_2-values are confirmed, base-line recordings of arterial blood gas results, RR, EIT-measurements and hemodynamic parameters are collected. Participants will thereafter be placed in the prone position with the bed at 0 degrees angle. Pillows are allowed for comfort. The measurements will be repeated at 30 and 60 minutes after start of PP. This is followed by a second semi-recumbent positioning as above with repeated measurements after 30 minutes. Flow rate and FiO_2 will remain unchanged during the study period unless desaturation < 90% for more than 5 min, or < 85% for more than 1 min occurs. Dyspnea assessed with the modified Borg-scale[20] will be recorded at baseline, after 30 and 60 min in PP and after 30 min in second semirecumbent positioning.

6. RANDOMIZATION AND BLINDING

Patients will be randomized 1:1 in blocks of four at each center. The randomization outcome is obtained via a web-based service provided by the coordinating center. The patient, the treating physician/-s and other care providers will not be blinded to the study allocation. Assessors will be blinded during analysis.

6.1 Concealment mechanism

An on-line central randomization service will be used.

7. OUTCOMES

7.1 Primary Outcome

The primary outcome is the rate of intubation.

7.2 Secondary outcomes

- time in PP/day in awake PP if measured. This will vary at different sites and wards.
- cross-over rate to NIV,
- incidence and days of vasopressor and/or inotropic support,
- incidence and days of renal replacement therapy,
- days on ventilator for patients in need of mechanical support,
- number of patients receiving extracorporeal membrane oxygenation,
- in-hospital and ICU LOS,
- complications.
- the 7- and 30-day mortality.
- WHO ordinal scale (Fig 1) for clinical improvement at baseline, day 7 and 30.

Fig 1. WHO ordinal scale for clinical improvement

Patient State	Description	Score
Uninfected	No clinical or virological evidence of infection	0
Ambulatory	No limitations of activities	1
	Limitations of activities	2
Hospitalized		
Mild disease	No oxygen therapy	3
	Oxygen by prongs or mask	4
Severe disease	NIV or HFNC	5
	Intubation and mechanical ventilation	6

	Mechanical ventilation +additional organ support (pressors, RRT, ECMO)	7
Dead	Death	8

7.2 Other parameters

Age, sex, weight, height, comorbidities and last available PaO_2/FiO_2 ratio before inclusion OR if arterial gas is not available, SpO_2/FiO_2 ratio will be recorded. Also, at baseline, viral diagnostic status, pulmonary radiology, available laboratory results regarding inflammation, anaemia, renal and hepatic function and biomarkers of cardiac dysfunction will be recorded.

7.3 EIT outcomes

In the subset of participants in the intervention group undergoing EIT measurements, further secondary outcomes at previously predefined timepoints are EELI, TIV, PaO_2/FiO_2 ratio, SpO_2, respiratory rate, blood pressure, heart rate and dyspnea assessed with the modified Borg-scale.

8. STATISTICAL METHODS

8.1 Sample size estimation

Sample size calculations are based on the ratio of intubation in critically ill COVID-19 patients in New York and Lombardy[17,18]. Assuming an intubation rate of 88% in the control group and a decrease of intubations of 20% in the intervention group a sample size of 112 patients in each group is needed (power 90%, alfa 0.05). To compensate for a drop out of 10%, 240 patients are planned for inclusion.

8.2 Interim analyses

Due to the lack of previous study data and the difficulties in predicting the course of the epidemic in Sweden, we plan to conduct interim analyses after inclusion of 120 and 180 patients. The decision to terminate the study may be based on 1) futility (lack of patients fulfilling the inclusion criteria), 2) safety (unexpected increase in the rate of intubation in the intervention group or increased occurrence of severe or unexpected adverse events, or 3) efficacy (if the reduction in the rate of intubation is more than 40%).

8.3 Statistical analysis

Intention-to-treat analyses will be conducted based on available data. The difference in rate of intubation between the groups will be analyzed with the Chi-2 test. Continuous data will be compared using multifactorial repeated measures ANOVA or multiple regression. Mean values with 95% confidence intervals will be calculated for primary and secondary outcomes. Non-parametric tests will be used for non-normally distributed data, with descriptive data expressed as medians with interquartile range.

9. ADVERSE EVENTS

All adverse (unfavorable, negative or harmful) events that are related to or possibly related to PP are to be reported by each participating center by the site investigator to the principal investigator on a case specific reporting form and classified as serious if it:

- Results in death
- Is life-threatening
- Requires admission to a higher level of care
- Requires medical or surgical interventions to prevent a more serious adverse event

All serious adverse events will be reported to the trial steering committee within 24 hours of occurrence.

Possible adverse event may be:

- Pressure wounds
- Aspiration
- Cardiac arrest

10.PRONE META-TRIAL

Data will be shared for meta-analysis of the effect of prone positioning in Covid-19 patients with the PRONE group[21,22], based on six multicenter randomised controlled trials with almost identical design, started in parallel during the first wave of the pandemic. A Data Transfer Agreement shall be signed before transfer of data.

REFERENCES

1. Coronavirus disease (COVID-2019) situation reports. Situation report -100

   (https://www.who.int/emergencies/diseases/novel-coronavirus-2019/situation-reports)

2. Huang C Wang Y Li X et al.Clinical features of patients infected with 2019 novel coronavirus in Wuhan, China. Lancet. 2020; 395: 497-506

3. Rochwerg B, Granton D, Wang DX, Helviz Y, Einav S, Frat JP, et al. High flow nasal cannula compared with conventional oxygen therapy for acute hypoxemic respiratory failure: a systematic review and meta-analysis. Intensive Care Med. 2019;45(5):563–572.

4. Frat JP, Thille AW, Mercat A, Girault C, Ragot S, Perbet S, Prat G, Boulain T, Morawiec E, Cottereau A, et al. High-flow oxygen through nasal cannula in acute hypoxemic respiratory failure. N Engl J Med. 2015;372(23):2185–96.
5. Messika J, Ben Ahmed K, Gaudry S, Miguel-Montanes R, Rafat C, Sztrymf B, Dreyfuss D, Ricard JD. Use of high-flow nasal cannula oxygen therapy in subjects with ARDS: a 1-year observational study. Respir Care. 2015;60(2):162–9.
6. Corley A, Caruana LR, Barnett AG, Tronstad O, Fraser JF. Oxygen Delivery Through High-Flow Nasal Cannulae Increase End-Expiratory Lung Volume and Reduce Respiratory Rate in PostCardiac Surgical Patients. Br J Anesth 2011: 107; 998-1004.
7. Riera J, Pérez P, Cortés J, Roca O, Masclans JR, Rello J. Effect of high-flow nasal cannula and body position on end-expiratory lung volume: a cohort study using electrical impedance tomography. Respir Care 2013; 58: 589-96.
8. Groves N, Tobin A. High flow nasal oxygen generates positive airway pressure in adult volunteers. Aust Crit Care. 2007; 20: 126-31.
9. Parke R, McGuinness S, Eccleston M. Nasal high-flow therapy delivers low level positive airway pressure. Br J Anaesth. 2009; 103: 886-90.
10. Ritchie JE, Williams AB, Gerard C. Evaluation of a high flow nasal oxygenation system: gas analysis and pharyngeal pressures. Intensive Care Med. 2011; 39: 1103-10
11. Gattinoni L, Tognoni G, Pesenti A, Taccone P, Mascheroni D, Labarta V, Malacrida R, Di Giulio P, Fumagalli R, Pelosi P, et al. Effect of prone positioning on the survival of patients with acute respiratory failure. N Engl J Med. 2001;345(8):568–73.
12. Guerin C, Gaillard S, Lemasson S, Ayzac L, Girard R, Beuret P, Palmier B, Le QV, Sirodot M, Rosselli S, et al. Effects of systematic prone positioning in hypoxemic acute respiratory failure: a randomized controlled trial. JAMA. 2004;292(19):2379–87.
13. Guerin C, Reignier J, Richard JC, Beuret P, Gacouin A, Boulain T, Mercier E, Badet M, Mercat A, Baudin O, et al. Prone positioning in severe acute respiratory distress syndrome. N Engl J Med. 2013;368(23):2159–68.
14. Munshi L, Del Sorbo L, Adhikari NKJ, Hodgson CL, Wunsch H, Meade MO, Uleryk E, Mancebo J, Pesenti A, Ranieri VM, et al. Prone position for acute respiratory distress syndrome. A systematic review and meta-analysis. Ann Am Thorac Soc. 2017;14(Supplement_4):S280–8.
15. Scaravilli V, Grasselli G, Castagna L, Zanella A, Isgrò S, Lucchini A, et al. Prone positioning improves oxygenation in spontaneously breathing nonintubated patients with hypoxemic acute respiratory failure: A retrospective study. J Crit Care. 2015;30:1390–4.
16. Ding L et al. Efficacy and Safety of Early Prone Positioning Combined with HFNC or NIV in Moderate to Severe ARDS: A Multi-Center Prospective Cohort Study. Crit Care 2020;24:114.
17. Grasselli G, Zangrillo A, Zanella A, Antonelli M, Cabrini L et al. Baseline Characteristics and Outcomes of 1591 Patients Infected With SARS-CoV-2 Admitted to ICUs of the Lombardy Region, Italy. JAMA, 2020, 323; 1574-8
18. Richardson S, Hirsch JS, Narasimhan M, Crawford JM, McGinn T et al. Presenting Characteristics, Comorbidities, and Outcomes Among 5700 Patients Hospitalized With COVID-19 in the New York City Area. JAMA, 2020, E-pub ahead of print.
19. Lobo B, Hermosa, C, Abella A et al. Electrical impedance tomography. Annals of Translational Medicine. 2018; 6: 26
20. Wilson RC, Jones PW. A comparison of the visual analogue scale and modified Borg scale for the measurement of dyspnea during exercise. Clin Sci, 1989, 76; 277-82

21. Awake prone positioning of hypoxaemic patients with COVID-19: protocol for a randomised controlled open-label superiority meta-trial. Tavernier E, McNicholas B, Pavlov I, Roca O, Perez Y, Laffey J, Mirza S, Cosgrave D, Vines D, Frat JP, Ehrmann S, Li J. BMJ Open. 2020 Nov 11;10(11)
22. Meta-trial of awake prone positioning with nasal high flow therapy: Invitation to join a pandemic collaborative research effort. Li J, Pavlov I, Laffey JG, Roca O, Mirza S, Perez Y, McNicholas B, Cosgrave D, Vines D, Tavernier E, Ehrmann S. J Crit Care. 2020 Dec;60:140-142. 21. Lobo B, Hermosa, C, Abella A et al.
