# Supplementary material for: Lung impedance changes during awake prone positioning in COVID-19. A non-randomized cross-over study
Source: PLoS One. 2024 Feb 21;19(2):e0299199. doi: 10.1371/journal.pone.0299199 (PMC10880988; doi:10.1371/journal.pone.0299199)
Supplement: S1 Table — (DOCX) [file pone.0299199.s003.docx]

**Table S3. Outcome variables of the study cohort at supine baseline, during prone positioning and after supine repositioning.**

| **Variable** | **T1** | **T2** | **T3** | **T4** |
| --- | --- | --- | --- | --- |
| TIV_Global_ (AU) | 100 (ref) | 93 (78 – 135) | 101 (79 – 108) | 96 (78 – 116) |
| TIV_Regional_ (%_TIVglobal_) |  |  |  |  |
| TIV_D_ | 18 (14 – 25) | 16 (15 – 23) | 16 (15 – 23) | 17 (13 – 21) |
| TIV_MD_ | 42 (39 – 45) | 43 (38 – 46) | 43 (38 – 47) | 43 (37 – 43) |
| TIV_MV_ | 33 (27 – 38) | 33 (29 – 36) | 33 (30 – 36) | 34 (28 – 41) |
| TIV_V_ | 6 (4 – 9) | 6 (3 – 11) | 6 (3 – 11) | 7 (6 – 11) |
| CoV (%) | 51 (46 – 59) | 50 (44 – 54) | 50 (44 – 54) | 52 (48 – 58) |
| dEELI_Global_ | 0 (ref) | 1.51 (0.25 – 4.03) | 1.51 (0.11 – 4.02) | 0.31 (-0.16 – 1.56) |
| dEELI_Regional_ (n=8) |  |  |  |  |
| dEELI_D_ | 0 (ref) | 0.49 (-0.10 – 0.77) | 0.54 (-0.03 – 0.87) | 0.14 (0.07 – 0.28) |
| dEELI_MD_ | 0 (ref) | -0.43 (-0.91 – 1.20) | -0.72 (-4.08 – 1.10) | -0.01 (-0.28 – 0.20) |
| dEELI_MV_ | 0 (ref) | 0.60 (-0.03 – 1.01) | 0.51 (-0.04 – 1.05) | -0.32 (-0.65 – -0.04 |
| dEELI_V_ | 0 (ref) | 0.25 (0.03 – 0.84) | 0.24 (-0.05 – 0.75) | 0.04 (-0.09 – 0.17) |
| GI-index | 51 (43 – 55) | 50 (45 – 58) | 48 (46 – 55) | 48 (42 – 54) |
| PaO_2_/FiO_2_ (kPa) | 18.9 (14.5 – 22.0) | 24.9 (16.5 – 26.2) | 23.6 (15.4 – 26.0) | 17.8 (14.5 – 24.3) |
| SpO_2_ (%) | 94 (93 – 95) | 97 (95 – 99) | 97 (95 – 98) | 94 (93 – 95) |
| RR (min^-1^) | 31 (20 – 36) | 27 (15 – 34) | 29 (20 – 34) | 31 (21 – 35) |
| PaCO_2_ (kPa) | 4.54 (3.82 – 4.69) | 4.53 (3.94 – 4.91) | 4.33 (3.88 – 4.90) | 4.15 (3.62 – 4.60) |
| Heart rate (min^-1^) | 66 (53 – 77) | 60 (51 – 75) | 62 (55 – 84) | 66 (59 – 77) |
| MAP (mmHg) | 80 (71 – 87) | 81 (73 – 90) | 88 (79 – 90) | 87 (65 – 97) |

Data are presented as median (interquartile range). EIT, electrical impedance tomography. T1-4: predefined data collection timepoints. T1, Supine baseline. T2, 30 min after prone positioning. T3, 60 min after prone positioning. T4, 30 min after supine repositioning. TIV, tidal impedance variation. CoV, Center of ventilation. dEELI, delta end-expiratory lung impedance. GI-index, global inhomogeneity index, RR, respiratory rate. MAP, mean arterial pressure. D, dorsal. MD, mid-dorsal. MV, mid-ventral. V, ventral.
