# Supplementary material for: Lung impedance changes during awake prone positioning in COVID-19. A non-randomized cross-over study
Source: PLoS One. 2024 Feb 21;19(2):e0299199. doi: 10.1371/journal.pone.0299199 (PMC10880988; doi:10.1371/journal.pone.0299199)
Supplement: S1 Fig — (PDF) [file pone.0299199.s004.pdf]

Fig S4. Scatterplot of correlation between change of PaO2/FiO2 ratio and change of dEELI from T1 (baseline, supine position) to T3 (60 min after prone positioning).

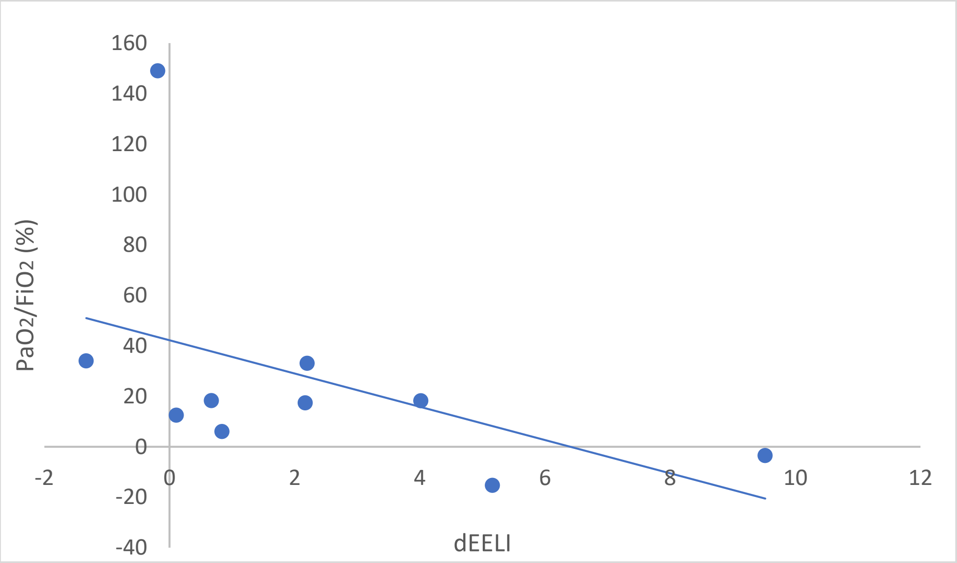

Spearman correlation analysis was used. Rho= -0.66, p=0.04 (Fig S4). dEELI, delta end-expiratory lung impedance.
